# Microbial, physicochemical and proximate analysis of Tej collected from Amhara regional state of Ethiopia

**DOI:** 10.1016/j.heliyon.2023.e16911

**Published:** 2023-06-01

**Authors:** Meseret Berhanu, Asnake Desalegn, Dagim Jirata Birri, Mogessie Ashenafi, Fitsum Tigu

**Affiliations:** a Department of Microbial, Cellular and Molecular Biology, College of Natural and Computational Science, Addis Ababa University, Ethiopia; b Center for Food Security Studies, College of Development Studies, Addis Ababa University, Ethiopia

**Keywords:** Alcoholic beverage, Ethiopian honey wine, Maturity time, Microbial quality, Tej fermentation

## Abstract

Tej is an Ethiopian traditional alcoholic beverage with significant social and economic importance. Due to the spontaneous fermentation process of Tej, several issues such as safety, quality, and physicochemical properties of the final products is rquired to be assessed. Thus, this study was aimed to assess the microbial quality, physicochemical, and proximate properties of Tej associated with different maturity time. The microbial, physicochemical and proximate analyses were carried out by standard protocol. Lactic acid bacteria (6.30 log CFU/mL) and yeast (6.22 log CFU/mL) were the dominat microorganisms of all Tej samples at different maturity time, with significant differences (p = 0.001) in mean microbial count among samples. The mean pH, titratable acidity and ethanol content of Tej samples were 3.51, 0.79 and 11.04% (v/v), respectively. There were significant differences (p = 0.001) among the mean pH and titratable acidity values. The mean proximate compositions (%) of Tej samples were as follows: moisture (91.88), ash (0.65), protein (1.38), fat (0.47) and carbohydrate (3.91). Statistically significant differences (p = 0.001) were observed in proximate compositions of Tej samples from different maturity time. Generally, Tej maturity time has a great impact on the improvement of nutrient composition and the increment of the acidic contents which in turn suppress the growth of unwanted microorganisms. Further evaluation of the biological, and chemical safety and development of yeast-LAB starter culture are strongly recommended to improve Tej fermentation in Ethiopia.

## 1 Introduction

Tej (Ethiopian honey wine) is a home-made and traditionally fermented product of Ethiopia. Traditionally prepared from mixtures of honey, water, the leaves, and the stems of *Rhamnus prinoides* (*Gesho*). Unknown microorganisms that originate from the raw materials or the utensils are responsible for the fermentation process [1]. Tej is categorized as an alcoholic beverage with a mean alcohol content of 7.0–10.9% (v/v). Varieties of honey wine have been produced in every corner of the country, such as Ogol [2], Booka [3], and Grawa [4]. However, Tej [5] is the most widely consumed product of honey (70–90% of honey produced per annum) by the majority of the population [6,7]. It has also significant social, cultural and economic importance and is considered as a national drink. It served on several social events such as marriage, social gatherings and spiritual holidays [7,8]. Besides this, it is an important source of nutrition due to the constitution of carbohydrate, protein and fat, and thus substantially supplements the people's daily diet [4,7,9].

**Fig. 1:**
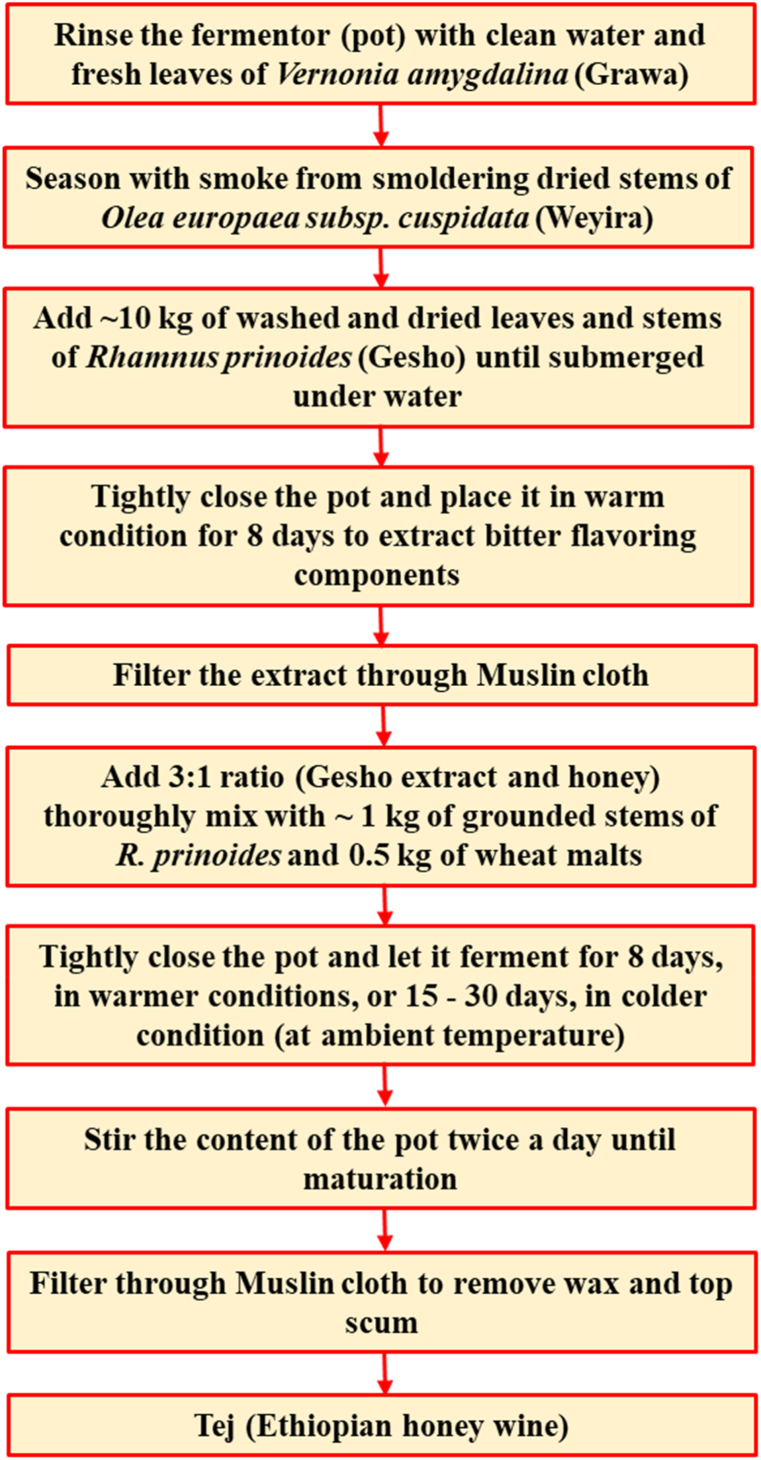
Flow chart for preparation of traditional Tej in Amhara region, Ethiopia (all amount of ingredients indicated in the flow chart is for one barrel).

**Fig. 2:**
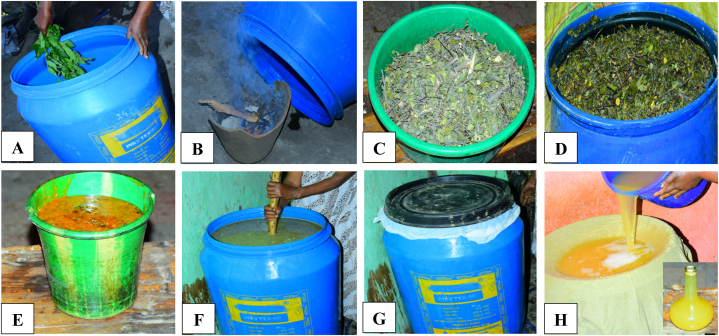
Illustration of a traditional Tej fermentation process. A) Pot cleaning with water and fresh leaves of *Vernonia amygdalina* (Grawa), B) Fumigation of pot by dried stems of *Olea europaea* subsp. *cuspidata* (Weyira), C) Dried leaves and stems of *Rhamnus prinoides* (Gesho), D) Extraction of bitter flavoring components by submerge state, E) Red honey, F) Fermenting wort (mixtures of honey, Gesho extracts, grounded stems of *R. prinoides* and wheat malts), G) Tightly close the pot and let it ferment (at ambient temperature), H) Filtration through Muslin cloth to remove sediment and *R. prinoides*, and Ready-to-drink Tej (small bottle with Tej, right bottom corner). (For interpretation of the references to color in this figure legend, the reader is referred to the Web version of this article.)

Tej is primarily produced from mixtures of honey, water, the leaves, and the stems of *Gesho*. However, there is a slight variation in the fermentation skills and raw materials used to make Tej among producers. In Amhara region Tej is prepared from a mixture of specific honey (red and multiflora honey), water extracts of leaves and stems of *Gesho*, and grounded wheat malts (Fig. 1, Fig. 2A–G). The whole fermentation process takes about 15–30 days depending on the weather condition. The final product has yellow color, sweet-sour alcoholic flavor, and effervescent and cloudy appearances with residual yeast cells, unfermented substrates, and other microorganisms (Fig. 2H) [9]. However, the flavor of Tej depends upon the areas from which the bees have collected the nectar and the climatic conditions of the environments [10].

Thus, the physicochemical properties of Tej may vary due to different factors. The processing practices including duration of fermentation and agro-ecological conditions of the honey contributed to the variations [11]. Honey is one of the major substrates used for the production of Tej. It has a recognized biological activity, the chemical composition of which depends on the floral origin, climatic, environmental and seasonal conditions, as well as on agricultural practices [12,13]. Honey contains about 200 different substances, with the main constituents being fermentable carbohydrates and minor components of minerals, proteins, vitamins, lipids, organic acids, amino acids, aroma compounds, flavonoids, phenolic acids, 5-hydroxymethylfurfural (MHF) pigments, waxes, pollen grains, enzymes and other phytochemicals [14]. Due to such important nutritional composition, honey is considered as the healthiest substrate for preparation of traditional fermented homemade drinks, including honey wine and fruit-honey wine [15].

Microbial fermentation makes the fermented beverage palatable as there will be an improvement in the organoleptic properties, texture, aroma, and flavor [16,17]. LAB and yeasts are the major producers of Tej and their metabolic products contribute to acidity and add a distinctive flavor and aroma to the fermenting material [11]. LAB belonging to the genus *Lactobacillus* isolated from Tej produces organic acids and diverse groups of antimicrobial agents, which are responsible for the upkeep quality and the palatability of fermented foods [18,19]. Yeasts belonging to the genus *Saccharomyces* were reported to be responsible for the conversion of sugars to ethanol in Tej [9,16].

The higher undesirable microbial counts in traditional alcoholic beverages indicated poor keeping quality of alcoholic beverages. Even though LAB are the most desirable microbes for Tej making the overabundance of LAB in the product could spoil the product faster due to over acidiﬁcation of the ﬁnal product within a short period of serving [20]. Lemi W, 2020 reported that the number of live microorganisms in traditional beverages contributed to the product's sourness within a few hours of serving [21]. The potential sources of the aerobic spores forming bacteria (ASFB) were associated with the addition of spices in the fermenting beverages and the raw materials used for fermentation [1]. Even though ASFB are unable to grow and multiply in the presence of lactic acid produced from LAB [22], they are the potential concerns of safety and stability of the beverages [23].

Due to the spontaneous fermentation process of Tej and the absence of national standards on the final product, issues such as microbial and chemical safety, quality, and physicochemical properties are required to be assessed. There is limited research information on the microbiological and physicochemical properties of Tej samples obtained from local retailers in Jimma, Addis Ababa, Bahir Dar, and Debre Markos provinces of Ethiopia [5,9,24]. However, to the best of our knowledge, there is a dearth of information in the literature particularly research focused on Tej maturity time. Besides, the microbial, physicochemical and proximate composition of Tej produced in Amhara region of Ethiopia have not been reported so far. Thus, this study was aimed to assess the microbial quality, physicochemical properties, and proximate compositions of Tej associated with different maturity time.

## 2 Materials and methods

### 2.1 Description of the study area

**Fig. 3:**
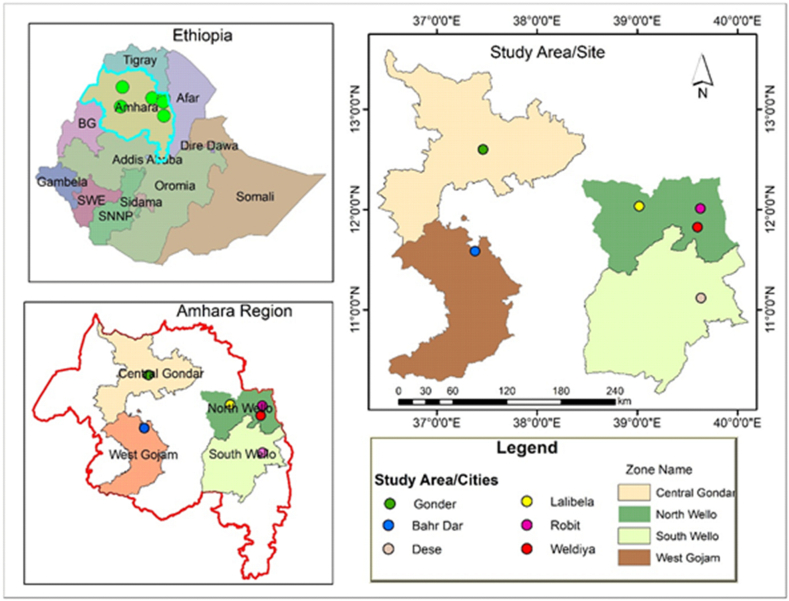
Map of the study area. The study country, Ethiopia (left corner), the Amhara Regional State (left bottom), the selected study areas (right corner) and the figure legend (right bottom).

This study was conducted in the Amhara regional state of Ethiopia including Bahir Dar, Gondar, Lalibela, Woldiya, and Dessie. Bahir Dar is the capital city and administrative center of the Amhara regional state. The city is located in the northwest of Ethiopia at a distance of 565 km away from the capital city of Ethiopia, Addis Ababa. Bahir Dar is located 11° 29′N latitude and 37° 29′ E longitudes, with an elevation of 1730 m above sea level (Fig. 3). It receive the highest rainfall (1150 mm) during June to September. It has an average temperature of 23 °C [25]. Gondar, Lalibela, Woldiya, and Dessie zones are located at 726, 676, 520, and 540 km from Addis Ababa, respectively. The areas receive the maximum amount of rainfalls ranging from 1016.9 to over 1592.0 mm annually and the average daily temperature of 21.5 °C.

### 2.2 Sample collection

A total of 30 Tej samples (five samples from each site), 1000 mL of each at three different maturity days, 10th day (soft and sweet-Tej), 15th day (moderately soft and sweet-Tej) and 30th day (hard and sweet-sour-Tej) were collected from Tej vending houses using purposive sampling techniques during January to February 2021. The samples were collected aseptically in new pre-sterilized plastic bottles and transported to Microbiology Laboratory, Addis Ababa University using an icebox. Samples requiring an instant analysis were performed immediately and the remains were stored at −20 °C.

### 2.3 Microbial analysis

The microbiological analysis of Tej samples such as LAB, yeast, aerobic mesophilic bacteria (AMB) and aerobic spore former bacteria (ASFB), and *Enterobacteriaceae*, were done by following the previously reported procedures [1,4]. Twenty-five milliliters of each sample was mixed with 225 mL of 0.1% (w/v) sterile peptone water and serially diluted for plating.

**2.3.1. Aerobic mesophilic bacteria (AMB)**: A volume of 0.1 mL aliquot from appropriate dilution was spread plated on pre-dried nutrient agar and incubated at 32 °C for 48 h. Colonies grown on the duplicate plates were counted as AMB.

**2.3.2. Coliform**: A volume of 0.1 mL aliquot from appropriate dilution was spread plated on pre-dried MacConkey agar (MAC) and incubated at 32 °C for 48 h. All colonies with red or pink colors were counted as total coliform.

**2.3.3. *Enterobacteriaceae***: A volume of 0.1 mL aliquot from appropriate dilution was spread-plated on pre-dried Violet Red Bile Dextrose Agar and incubated at 32 °C for 24 h. Then, purple or pink colored colonies surrounded by purple halos were counted as members of *Enterobacteriaceae*.

**2.3.4. Aerobic spore-forming bacteria (ASFB)**: From appropriate dilution, 10 mL was heat-treated at 80 °C of water bath for 10 min to kill the vegetative cells and cooled by gentle flowing water. Then, a volume of 0.1 mL aliquot from appropriate dilution was spread-plated on pre-dried nutrient agar plates and incubated at 32 °C for 48 h.

**2.3.5. Lactic acid bacteria (LAB)**: A volume of 0.1 mL aliquot from appropriate dilution was spread plated on pre-dried de Man, Rogosa and Sharpe agar (MRS) plates and incubated anaerobically at 30–32 °C for 48 h using anaerobic Jar (BD BBL, Gas Pak Anaerobic System).

**2.3.6. Yeast and mold**: A volume of 0.1 mL aliquot from appropriate dilution was spread-plated on pre-dried Rose Bengal agar plates supplemented with 0.1 g/l chloramphenicol and incubated at 25–28 °C for 2–5 days. Smooth, non-hairy colonies lacking extensions at margins under stereoscopic microscope were counted as yeasts and while either colonies were counted as mold.

### 2.4 Physicochemical parameters

The pH of the Tej sample was measured using a digital portal pH meter (pH-013, China) as the protocol indicated elsewhere [9]. The titratable acidity was determined by measuring 10 mL of Tej sample and directly pipetting into a conical flask and titrating against 1.0 M NaOH and phenolphthalein as an indicator [26]. While the specific gravity method of AOAC was used to measure the samples' total alcohol content [27].

### 2.5 Proximate analysis

The proximate composition of the Tej samples were determined by the AOAC method [27]. Fifty milliliters of Tej sample was oven-dried at 105 °C for 2 h and the moisture content was determined. Ash content was determined by igniting a 5 mL sample added to the crucible and placed in a muﬄe furnace at 550 °C for 4 h and allowed to cool in a desiccator and ash content was calculated. The Kjeldahl method was followed to determine the total protein content. While total fat and total carbohydrate contents were determined by the standard protocols indicated in AOAC.

### 2.6 Statistical analysis

All data sets were analyzed by SPSS version 26.0 software. Descriptive statistics was employed to identify the mean, percentage, and standard deviation. Pearson correlation, regression, and ANOVA were used in the inferential statistics for quantitative data. *P*-value ≤0.05 was considered statistically significant.

## 3 Results

### 3.1 Microbial enumeration

The results of microbial enumeration of Tej samples collected at different maturity time from Amhara regional state were summarized in Table 1 and S Table 1. The mean count (log CFU/mL) indicated that LAB and yeast were dominantly found in all samples of Tej and all maturity time. It ranged from 6.26 ± 0.07 to 6.37 ± 0.02 and 6.16 ± 0.11 to 6.34 ± 0.04, respectively. Among the samples, the highest mean counts (6.37 ± 0.02 and 6.34 ± 0.04) of LAB and yeast were recorded in one-month old Tej samples, respectively. The least counts of LAB (6.26 ± 0.07) and yeast (6.16 ± 0.11) were recorded at the 10^th^ and 15^th^ time of Tej samples, respectively. AMB and ASFB counts (log CFU/mL) ranged from 4.71 ± 0.07 to 4.86 ± 0.11 2.79 and 2.64 ± 0.09 to 2.69 ± 0.04, respectively from different maturity days. While in all samples of Tej, the coliform bacteria, *Enterobacteriaceae* and molds were not detected (below the detection levels). With the exception of ASFB counts, statistically significant differences (p < 0.001) were observed among all other microbial counts with different maturity days of Tej. However, there are no statistically significant differences in microbial counts among the six Tej sampling sites of Amhara region (S Table 2).

**Table 1:** Microbial profile (log CFU/ml) of Tej at different maturity time (day) collected from Amhara Region, Ethiopia.

Maturity time	N	AMB	ASFB	LAB	Yeast	Mold
10	12	4.83 ± 0.05	2.69 ± 0.04	6.26 ± 0.07	6.16 ± 0.11	<2
15	12	4.86 ± 0.11	2.64 ± 0.09	6.27 ± 0.08	6.16 ± 0.09	<2
30	6	4.71 ± 0.07	2.67 ± 0.04	6.37 ± 0.02	6.34 ± 0.04	<2
p-value	–	0.006	0.191	0.018	0.001	–

Microbial count data (Mean ± SD), Maturity time (day), p-value <0.05 considered as statistically significant differences in mean microbial counts among different maturity time of Tej samples, AMB = Aerobic mesophilic bacteria, ASFB = Aerobic spore-forming bacteria, LAB = Lactic acid bacteria.

### 3.2 Physicochemical and proximate analyses

The pH values of Tej samples collected at three different maturity days varied between 3.29 ± 0.15 and 3.73 ± 0.17 (Table 2). The variation of pH values among the three different maturity days were statistically significant at p-value = 0.001. Similarly, the values of titratable acidity among the three different maturity days varied from 0.42 ± 0.11 to 1.16 ± 0.35 with significant difference at p-value = 0.001. The alcohol content (% v/v) of Tej samples varied between 10.07 ± 0.71 and 12.00 ± 0.85. Significant variation was observed among the three different maturity times (p = 0.001). But there are no statistically significant differences among the samples of the six sampling sites (p = 0.29).

**Table 2:** Physicochemical and proximate profile of Tej at different maturity time (day) collected from Amhara Region, Ethiopia.

Maturity time	N	Physicochemical and proximate composition							
		pH	TA	Ethanol	Moisture	Ash	Protein	Fat	Carbohydrate
10	12	3.73 ± 0.17	0.42 ± 0.11	10.07 ± 0.71	97.04 ± 0.88	0.36 ± 0.14	0.58 ± 0.32	0.25 ± 0.11	5.58 ± 2.89
15	12	3.50 ± 0.12	0.78 ± 0.15	11.05 ± 0.60	93.64 ± 2.03	0.72 ± 0.07	1.24 ± 0.35	0.54 ± 0.19	3.77 ± 1.17
30	6	3.29 ± 0.15	1.16 ± 0.35	12.00 ± 0.85	84.97 ± 0.70	0.88 ± 0.07	2.32 ± 0.87	0.63 ± 0.18	2.39 ± 1.24
p-value	–	0.001	0.001	0.001	0.001	0.001	0.001	0.001	0.001

All data (Mean ± SD), Maturity time (day).

The proximate composition analysis indicated that there were statistically significant differences (p < 0.05) among the three maturity days of Tej samples (Table 2). Accordingly, the mean minimum (84.97 ± 0.70) and mean maximum (97.04 ± 0.88) moisture content was recorded for the 30^th^ and 10^th^ days Tej samples, respectively. Likewise, the protein and fat contents (g/100 mL) has varied with maturity days, with mean minimum (0.58 ± 0.32 and 0.25 ± 0.11) and mean maximum (2.32 ± 0.87 and 0.63 ± 0.18) protein and fat contents were recorded at 10^th^ and 30^th^ days of Tej samples, respectively. Furthermore, mean ash content of Tej sample varied with the maturity days, accordingly the least (0.36 ± 0.14) and highest (0.88 ± 0.07) ash content was recorded at 10^th^ and 30^th^ days of Tej samples, respectively. With regard to the mean total carbohydrate content (g/100 mL) of Tej samples, it also varied from 5.58 ± 2.89 and 2.39 ± 1.24 in 10^th^ and 30^th^ days of maturity time, respectively. Statistically significant variation was observed among the samples with maturity time and sampling sites (p < 0.001). Overall, protein, fat and ash content has increased with an increase of Tej maturity days with the exception of moisture content and carbohydrate (S Table 3).

## 4 Discussion

In this study, over 30 Tej samples at three different maturity days from six major honey producing sites of Amhara regional state were considered for microbiological, physicochemical and proximate investigation. The results indicated that, the LAB and yeast populations were dominantly observed throughout maturity days of Tej samples. Several studies [1,24,28] also reported that LAB and yeast were the dominant microorganisms involved in Tej fermentation. In many fermentation processes, some group of microorganisms, particularly LAB and yeast species have synergistic activity towards the co-fermentation of the common substrate as reported by many researchers [[29], [30], [31]]. Recently, metagenomics study of spontaneously fermented Tej revealed the presence of several species of *Saccharomyces* and *Lactobacillus* [19,32]. The positive interactions of LAB and yeast species in the fermentation process results in the development of organoleptic, nutritional and safety of the final products of alcoholic beverages, including Tej [28].

However, the absence of coliform bacteria and *Enterobacteriaceae* may be associated with the production of antimicrobial substances such as organic acids, bacteriocins, and hydrogen peroxide by some LAB species which leads to the acidification of the fermentation broth [33]. Furthermore, the absence of indicator microorganisms are related to the traditional practices of Tej fermentation. During the traditional process of Tej fermentation, there is a long history among indigenous people in that the container is preconditioned by the two medicinal plants known as *Vernonia amygdalina* (Grawa) and *Olea europaea* subsp. *cuspidata* (Weyira). The first plant is used to clean the container, and the latter is used to treat the container by fumigation. Traditionally, these practices are used to reduce the pathogenic microorganisms and enhance the growth and proliferation of yeast and LAB that are involved in the fermentation of Tej. Although this finding is in agreement with that of Bahiru et al., [1] a significant number (≤3 log CFU/mL) of *Enterobacteriaceae* and other members of pathogenic microorganism were reported from Tej and other traditional beverages by other researchers [4,24]. The differences might be due to the variation in Tej fermentation skills of the society associated with personal hygiene, container cleaning and conditioning, the way of adding and treating ingredients and other additives [9].

The mean AMB count (>4.71 log CFU/mL) in this study is relatively higher than figures from other similar studies [1,4]. The high loads (>5 log cfu/mL) of AMB in many fermented beverages are unacceptable due to safety issues. Our study finding is in the ranges of the maximum permissible limits of AMB. Compared to other studies [1,4], the presence of AMB count in the wine samples could be due to inappropriate hygienic practices during the production, in particular poor handling and treatment of raw material, equipment used for processing, and use of poor quality water are among some reasons [34].

The values of pH and titratable acidity in this study showed significant variations with variable maturity times of Tej. The pH varied from 3.73 ± 0.17 at the 10^th^ day to 3.29 ± 0.15 at the 30^th^ day of maturity. The mean pH of Tej in our study is lower than that of beetroot wine, 3.20 to 3.37 [35], Booka wine, 3.01 [3], similar with the other reports [4,9]. However, it is higher than the reports of Fentie et al., 2022 (4.3) and other similar products of Africa, yellow wine of Cameroon (4.35–3.84) [36]. Similarly, the titratable acidity also increased with the increase of the maturity days of Tej. It varied from 0.42 ± 0.11 at the 10^th^ day to 1.16 ± 0.35 at the 30^th^ day of maturity. Higher titratable acidity was observed in this study as comparable with the study reported by other findings [4,35], but lower than the reports of Fentie et al. (2022) and Bayoi et al. (2021). Comparing the progressive increase of the titratable acidity with the microbial counts at different maturity times of Tej, except the AMB count, other microbes (ASFB, LAB, and yeast) did not show any decline in terms of number (S Table 1). A similar observation was reported that at the initial stages of fermentation (<10 days), LAB population showed a declined trend and after 20 days the population remained stable until the end of their experiments (50^th^ days) [37]. Likewise, Torija et al. [38], reported that the non-*Saccharomyces* yeasts were limited to early stages of fermentation of wines, whilst the *Saccharomyces* yeasts dominated towards the end of alcoholic fermentation. This is due to the fact that *Saccharomyces* yeast are more tolerant to ethanol and more competitive in a growth medium containing high concentrations of sugar [39].

Various factors are responsible for the physicochemical variations of honey wines including Tej. The processing practices including fermentation periods and agro-ecological conditions that honey is harvested [11], and accumulations of organic acids, particularly lactic acid from LAB and acetic acid originated from other contaminated microbes [19,22,33] are considered as major factors. Besides, the higher counts of LAB and yeast's secondary metabolites might contribute to the lowering of pH through the conversion of high sugar content of honey to alcohol, organic acids, and, CO_2_ by the action of microorganisms [40]. Titratable acidity and low pH have a direct reflection to the quality of alcoholic beverages or enhancing the overall characteristics and balance of the honey wine as maintaining good shelf stability as well as safety via inhibition to the growth of pathogenic microorganisms [28,[41], [42], [43]].

The other most important parameters of alcoholic beverages are ethanol concentration of the product. Since various yeast species are involved in the fermentation process of Tej, and their metabolic end product of which is mainly ethanol, thus quantification and comparison of the alcohol content of different samples of Tej are quite reasonable. In our study higher alcohol content and statistically significant differences in the alcohol content between samples and maturity time were observed. The mean alcohol content of Tej in this study is higher than that of Bahiru et al. [9] and Nemo & Bacha [4]. This variation could be due to the differences in the maturity time, the amount of substrate (honey) and additives and as well as the kind of yeast strains and other natural microflora originated from raw materials and equipment used. It has been logical that as the maturity time increases, the alcohol content of the beverages also increases. Thus, the same is true in our Tej sample that the 30^th^ day sample has higher alcoholic concentration than the earliest mature Tej (10^th^ day).

Physicochemical and proximate compositions have an impact on the safety and quality of beverages via reducing the microbial load and enhancing organoleptic properties [44]. In our study, the proximate composition of Tej was influenced by the maturity time. The moisture content of the Tej varied from 98.19 at the 10^th^ day of maturity to 81.25 at the 30^th^ day of maturity. The mean moisture content of Tej in our study is lower than that of other Tej and Booka samples collected from other parts of Ethiopia, and coconut wine in Africa, it ranges from 95.78 to 97.20 [3,4,45]. But it is higher than that of water melon wine, 70.94 [46] and banana wine, 67.20 [47]. The reduction of moisture content as the increments of maturity time might be due to the accumulation of organic matter having an opportunity to increase during fermentation as the result of microbial proliferation [48].

Another important proximate component in this study is the total ash content of Tej. It increased from 0.61 to 0.98 as the maturity time of Tej samples increased from 10^th^ days to 30^th^ days. The result showed that different maturity times of Tej samples were statistically significant (p = 0.001). This result indicated that the ash content of different Tej samples is affected by the maturity time, such phenomenon could contribute to the leaching of minerals through fermentation [49]. Higher ash content was observed in this study as compared to the study reported by Nemo and Bacha [4]. But it is lower than that of Booka wine [3], banana wine [47], beetroot wine [35]. Higher ash content leads to an increase in mineral content and it is an indication of the level of mineral composition of the substrates [50]. In all the samples of our study, high ash content was observed in the longest time of maturity. Ozabor et al. [38] also reported the same characteristics of ash content increment with an increase of maturity time.

With regard to crude protein content of our Tej sample, variable protein concentrations were reordered in different maturity time. The highest crude protein content was recorded for the one month aged Tej sample (2.32 ± 0.87) compared to ten days old Tej sample (0.58 ± 0.32) with statistically significant (p = 0.001) differences with maturity time. The mean crude protein content of Tej in our study is higher than similar studies conducted in other parts of Ethiopia [4], wine prepared from water melon [46], banana wine [47]. The higher crude protein content in the current study may be associated with the traditional Tej makers’ experience that they add some amount of fresh honey to the old Tej sample in order to increase the shelf-life and to reduce the strength of Tej particularly, the acidity (personal communication with the local Tej makers). Thus, the residues of larvae in the fresh honey might increase the protein content of Tej. The other possible reason for the higher crude protein content of Tej is due to microbial cell proliferation [43]. Fermentation may result in increasing total nitrogen content and free amino acids, and microbial metabolites or it can be due to proteolytic enzymes produced by the fermentative organisms [51].

The fat content of the Tej samples has also the same trends like other proximate components. As the maturity time increased, the fat content of Tej samples also increased, with significant differences (p = 0.001) among different maturity days. The fact that the higher total fat content is associated with the fermentation process degradation of the nutrient composition of the substrates as reported elsewhere [52]. Comparing our study findings with other similar studies, our mean total fat content is higher than that of the Tej samples collected from other parts of Ethiopia in a previous study [4] as well as related products in Africa such as water melon wine [46] and banana wine [47]. The current total fat content is much lower than that of the other reports [3,9,45]. However, contrary to these observations, a study has reported that the fermentation process has decreased the fat content of alcoholic beverages [50]. Thus, the discrepancies of such observation may be due to the differences in the fermentation skills, the substrates used to make honey wine and the kind of microorganisms involved in the lipid metabolism. But in this study, the addition of some fresh honey as it mentioned earlier is the main reason for the higher content of fat in the old samples of Tej.

The carbohydrate content of Tej samples reduced signiﬁcantly (p = 0.001) with increase in maturity time from 10^th^ day to 30^th^ days. The reduction of total carbohydrate concentration is as a result of the breakdown of sugar to ethanol and carbon dioxide, as the days of maturation progressed. This is due to the fact that fermentation facilitates the accessibility of carbohydrates, increases protein and fat concentrations, improves the bioavailability and accessibility of other nutrients and ultimately extends the shelf life of the product through the activation of endogenous enzymes [53]. The mean total carbohydrate content of our Tej samples is higher than that of the samples collected from other parts of Ethiopia [3,4,45]. The high concentration of carbohydrate at an early stage of Tej maturity could be serving as meal beverage replacement for the community.

## 5 Conclusion

In this study microbiological, physiochemical and proximate analyses of Tej samples collected in different maturity time from six different sites of the Amhara regional state of Ethiopia were evaluated. Microbiological point of view, the entire Tej samples tested in this study contained lesser or undetectable numbers of undesirable microorganisms. However, the fermentative microbes such as LAB and yeasts were dominantly found in every sample and maturity levels of Tej. We also observed the acidity, ash, protein and fat contents of Tej samples has continuously increased with an increase of the maturity time with the exceptions of moisture and carbohydrate. Overall, Tej maturity time has a significant impact on the physicochemical properties, proximate compositions and keeping quality of the final product. Tej fermentation and processing in the study areas totally depends on household skills, and thus the fermentation outcome is highly variable and unpredictable. Thus, making use of the most dominant fermentative microorganisms, LAB and yeasts as a mixed starter culture together with process optimization and standardization of the final product is highly recommended to commercialize Tej in Ethiopia. Finally, the authors also recommend the identification of microbiota and the role of microorganisms in the production of Tej.

## Authors contribution statement

Meseret Berhanu: Conceived and designed the experiments; Performed the experiments; Analyzed and interpreted the data; Wrote the paper.

Asnake Desalegn: Conceived and designed the experiments; Analyzed and interpreted the data; Contributed reagents, materials, analysis tools or data.

Dagim Jirata Birri: Conceived and designed the experiments; Contributed reagents, materials, analysis tools or data.

Mogessie Ashenafi: Conceived and designed the experiments.

Fitsum Tigu: Conceived and designed the experiments; Analyzed and interpreted the data; Contributed reagents, materials, analysis tools or data; Wrote the paper.

## Data availability

Data included in article/supplementary material/referenced in article.

## Funding information

This work was supported by the 10.13039/501100007941Addis Ababa University 7^th^ Round Thematic Research Fund [RD/LT076, 2019].

## Declaration of competing interest

Authors declare that there is no conflict of interest and all authors also read and approved the final manuscript for submission. We also certify that the article is the authors' original work hasn't received prior publication and isn't under consideration for publication elsewhere.
