# Supplementary material for: Microbial, physicochemical and proximate analysis of Tej collected from Amhara regional state of Ethiopia
Source: Heliyon. 2023 Jun 1;9(6):e16911. doi: 10.1016/j.heliyon.2023.e16911 (PMC10275989; doi:10.1016/j.heliyon.2023.e16911)
Supplement: Multimedia component 1 [file mmc1.docx]

**Microbial, physicochemical and proximate analysis of Tej collected from Amhara regional state of Ethiopia**

Meseret Berhanu^1^, Asnake Desalegn^1^, Dagim Jirata Birri^1^, Mogessie Ashenafi^2^, Fitsum Tigu^1,*^

^1^Department of Microbial, Cellular and Molecular Biology, College of Natural and Computational Science, Addis Ababa University

^2^ Center for Food Security Studies, College of Development Studies,

Addis Ababa University

**Supplemenatry files**

**Supplementary Table 1**: Mean microbial count (log CFU/ml) of Tej collected from different sampling sites and maturity time

| **Sample area** | **N** | **M. time*** | **AMB** | **ASFB** | **LAB** | **Yeast** |
| --- | --- | --- | --- | --- | --- | --- |
| Dessie | 2 | 10 | 4.82 ± 0.02 | 2.69 ± 0.05 | 6.13 ± 0.01 | 6.05 ± 0.03 |
|  | 2 | 15 | 4.86 ± 0.06 | 2.63 ± 0.15 | 6.24 ± 0.06 | 6.11 ± 0.03 |
|  | 1 | 30 | 4.64 ± 0.02 | 2.67 ± 0.04 | 6.33 ± 0.01 | 6.30 ± 0.01 |
| Woldiya | 2 | 10 | 4.85 ± 0.04 | 2.66 ± 0.03 | 6.36 ± 0.03 | 6.12 ± 0.10 |
|  | 2 | 15 | 4.78 ± 0.16 | 2.70 ± 0.03 | 6.35 ± 0.02 | 6.17 ± 0.14 |
|  | 1 | 30 | 4.62 ± 0.04 | 2.63 ± 0.01 | 6.40 ± 0.03 | 6.33 ± 0.01 |
| Robit | 2 | 10 | 4.82 ± 0.04 | 2.67 ± 0.01 | 6.34 ± 0.02 | 6.23 ± 0.03 |
|  | 2 | 15 | 4.98 ± 0.01 | 2.63 ± 0.15 | 6.10 ± 0.02 | 6.24 ± 0.02 |
|  | 1 | 30 | 4.74 ± 0.02 | 2.72 ± 0.01 | 6.38 ± 0.02 | 6.31 ± 0.01 |
| Lalibela | 2 | 10 | 4.81 ± 0.08 | 2.75 ± 0.04 | 6.27 ± 0.13 | 6.11 ± 0.09 |
|  | 2 | 15 | 4.98 ± 0.01 | 2.63 ± 0.10 | 6.30 ± 0.01 | 6.19 ± 0.06 |
|  | 1 | 30 | 4.70 ± 0.10 | 2.74 ± 0.04 | 6.36 ± 0.06 | 6.36 ± 0.01 |
| Gondar | 2 | 10 | 4.81 ± 0.01 | 2.69 ± 0.10 | 6.23 ± 0.08 | 6.13 ± 0.12 |
|  | 2 | 15 | 4.78 ± 0.01 | 2.70 ± 0.04 | 6.29 ± 0.00 | 6.19 ± 0.02 |
|  | 1 | 30 | 4.82 ± 0.01 | 2.68 ± 0.01 | 6.38 ± 0.04 | 6.39 ± 0.00 |
| Bahir Dar | 2 | 10 | 4.89 ± 0.01 | 2.75 ± 0.07 | 6.24 ± 0.12 | 6.35 ± 0.03 |
|  | 2 | 15 | 4.84 ± 0.01 | 2.70 ± 0.07 | 6.28 ± 0.08 | 6.19 ± 0.01 |
|  | 1 | 30 | 4.66 ± 0.02 | 2.65 ± 0.04 | 6.40 ± .000 | 6.40 ± 0.02 |

* Maturity time (day); Microbial count data (Mean ± SD), AMB = Aerobic mesophilic bacteria, ASFB = Aerobic spore-forming bacteria, LAB = Lactic acid bacteria.

**Supplementary Table 2**: Statistical comparison of mean microbial count (log CFU/ml) of Tej between various sampling sites

| **Sample area** | **N** | **Microbial loads (log CFU/ml)** | | | |
| --- | --- | --- | --- | --- | --- |
|  |  | **Yeast** | **LAB** | **AMB** | **ASFB** |
| Dessie | 5 | 6.12 ± 0.10 | 6.21 ± 0.08 | 4.82 ± 0.05 | 2.65 ± 0.09 |
| Woldiya | 5 | 6.17 ± 0.13 | 6.36 ± 0.02 | 4.77 ± 0. 13 | 2.66 ± 0.03 |
| Robit | 5 | 6.24 ± 0.03 | 6.25 ± 0.14 | 4.86 ± 0.11 | 2.65 ± 0.09 |
| Lalibela | 5 | 6.18 ± 0.11 | 6.29 ± 0.03 | 4.85 ± 0.12 | 2.69 ± 0.09 |
| Gondar | 5 | 6.22 ± 0.12 | 6.29 ± 0.06 | 4.79 ± 0.04 | 2.65 ± 0.06 |
| Bahir Dar | 5 | 6.24 ± 0.16 | 6.30 ± 0.06 | 4.80 ± 0.10 | 2.69 ± 0.05 |
| Total | 30 | 6.20 ± 0.11 | 6.28 ± 0.08 | 4.81 ± 0.09 | 2.67 ± 0.07 |
| p-value | - | 0.555 | 0.101 | 0.662 | 0.901 |

**Supplementary Table 3**: Physicochemical and proximate analysis of Tej collected from different sampling sites and maturity time

| **Sample area** | **N** | **M. time*** | **Physicochemical and proximate composition** | | | | | | | |
| --- | --- | --- | --- | --- | --- | --- | --- | --- | --- | --- |
|  |  |  | **pH** | **TA** | **Ethanol** | **Moisture** | **Ash** | **Protein** | **Fat** | **Carbohydrate** |
| Dessie | 2 | 10 | 3.62 ± 0.11 | 0.47 ± 0.01 | 9.63 ± 0.59 | 97.65 ± 0.04 | 0.43 ± 0.01 | 0.80 ± 0.01 | 0.10 ± 0.04 | 5.31 ± 1.78 |
|  | 2 | 15 | 3.57 ± 0.08 | 0.57 ± 0.01 | 10.97 ± 0.73 | 96.33 ± 0.06 | 0.66 ± 0.02 | 0.99 ± 0.02 | 0.36 ± 0.01 | 4.28 ± 1.35 |
|  | 1 | 30 | 3.40 ± 0.01 | 1.07 ± 0.06 | 11.50 ± 0.83 | 87.12 ± 0.01 | 0.94 ± 0.06 | 2.24 ± 0.10 | 0.43 ± 0.02 | 3.62 ± 1.79 |
| Woldiya | 2 | 10 | 3.56 ± 0.01 | 0.54 ± 0.00 | 10.39 ± 0.40 | 96.74 ± 0.07 | 0.59 ± 0.10 | 0.98 ± 0.02 | 0.20 ± 0.01 | 4.65 ± 0.43 |
|  | 2 | 15 | 3.46 ± 0.01 | 0.72 ± 0.00 | 11.03 ± 0.09 | 94.16 ± 0.13 | 0.70 ± 0.04 | 1.38 ± 0.08 | 0.35 ± 0.01 | 3.64 ± 0.44 |
|  | 1 | 30 | 3.35 ± 0.01 | 1.07 ± 0.08 | 11.84 ± 0.09 | 81.25 ± 0.67 | 0.91 ± 0.00 | 3.40 ± 0.33 | 0.52 ± 0.02 | 2.80 ± 0.31 |
| Robit | 2 | 10 | 3.66 ± 0.01 | 0.43 ± 0.04 | 10.39 ± 0.47 | 95.81 ± 0.05 | 0.22 ± 0.08 | 0.63 ± 0.04 | 0.27 ± 0.01 | 4.08 ± 0.99 |
|  | 2 | 15 | 3.61 ± 0.01 | 0.47 ± 0.04 | 11.12 ± 0.11 | 94.64 ± 0.12 | 0.79 ± 0.06 | 1.61 ± 0.01 | 0.39 ± 0.01 | 2.78 ± 0.44 |
|  | 1 | 30 | 3.12 ± 0.01 | 1.53 ± 0.00 | 11.81 ± 0.14 | 82.62 ± 0.01 | 0.88 ± 0.01 | 1.36 ± 0.06 | 0.51 ± 0.07 | 1.99 ± 1.39 |
| Lalibela | 2 | 10 | 3.84 ± 0.02 | 0.49 ± 0.02 | 10.76 ± 0.98 | 97.85 ± 0.09 | 0.60 ± 0.33 | 0.33 ± 0.06 | 0.38 ± 0.02 | 4.07 ± 0.80 |
|  | 2 | 15 | 3.67 ± 0.01 | 0.72 ± 0.01 | 11.49 ± 0.72 | 97.15 ± 0.06 | 0.74 ± 0.04 | 1.02 ± 0.04 | 0.81 ± 0.01 | 4.96 ± 0.75 |
|  | 1 | 30 | 3.17 ± 0.02 | 0.65 ± 0.02 | 11.93 ± 1.35 | 88.13 ± 0.01 | 0.84 ± 0.04 | 3.36 ± 0.06 | 0.79 ± 0.01 | 0.94 ± 0.22 |
| Gondar | 2 | 10 | 3.95 ± 0.02 | 0.28 ± 0.01 | 9.29 ± 0.22 | 96.48 ± 0.12 | 0.38 ± 0.01 | 0.20 ± 0.01 | 0.39 ± 0.01 | 4.69 ± 1.64 |
|  | 2 | 15 | 3.87 ± 0.02 | 0.37 ± 0.01 | 10.68 ± 0.91 | 95.56 ± 0.07 | 0.77 ± 0.02 | 1.13 ± 0.70 | 0.70 ± 0.02 | 3.20 ± 1.37 |
|  | 1 | 30 | 3.52 ± 0.01 | 0.55 ± 0.01 | 11.67 ± 0.80 | 86.42 ± 0.71 | 0.75 ± 0.06 | 1.58 ± 0.05 | 0.88 ± 0.01 | 2.40 ± 1.93 |
| Bahir Dar | 2 | 10 | 3.78 ± 0.01 | 0.32 ± 0.01 | 9.94 ± 0.38 | 97.76 ± 0.33 | 0.30 ± 0.05 | 0.55 ± 0.04 | 0.23 ± 0.04 | 10.67 ± 3.67 |
|  | 2 | 15 | 3.74 ± 0.01 | 0.52 ± 0.01 | 11.02 ± 0.63 | 96.98 ± 0.03 | 0.71 ± 0.01 | 1.38 ± 0.03 | 0.65 ± 0.01 | 3.78 ± 1.35 |
|  | 1 | 30 | 3.23 ± 0.01 | 1.26 ± 0.06 | 13.27 ± 0.86 | 84.33 ± 0.28 | 0.98 ± 0.01 | 2.03 ± 0.01 | 0.72 ± 0.01 | 2.61 ± 0.37 |

All data (Mean ± SD), Maturity time (day)
